# Bis{2-[bis­(3,5-dimethyl-1*H*-pyrazol-1-yl-κ*N* ^2^)meth­yl]pyridine-κ*N*}copper(II) dinitrate

**DOI:** 10.1107/S1600536811045636

**Published:** 2011-11-05

**Authors:** Jia-Cheng Liu, Guo-Zhe Guo, Chao-Hu Xiao, Xue-Yan Song, Meng Li

**Affiliations:** a Key Laboratory of Polymer Materials of Gansu Province, Key Laboratory of Bioelectrochemistry & Environmental Analysis of Gansu College of Chemistry and Chemical Engineering, Northwest Normal University, Lanzhou 730070, People’s Republic of China

## Abstract

In the mononuclear title complex, [Cu(C_16_H_19_N_5_)_2_](NO_3_)_2_, the Cu^II^ ion is located on a twofold rotation axis and is six-coordinated by six N atoms from two 2-[bis­(3,5-dimethyl-1*H*-pyrazol-1-yl)meth­yl]pyridine ligands, forming a distorted octa­hedral geometry. In the crystal, mol­ecules are linked by weak C—H⋯O inter­actions.

## Related literature

For background to complexes based on rigid ligands containing pyrazole, see: Zhang *et al.* (2009 ▶); Otten *et al.* (2009 ▶); Arroyo *et al.* (2000 ▶); Morin *et al.* (2011 ▶). For the bioinorganic chemistry of cooper complexes, see: Turski & Thiele (2009 ▶); Finney *et al.* (2009 ▶); Tardito & Marchiò (2009 ▶).
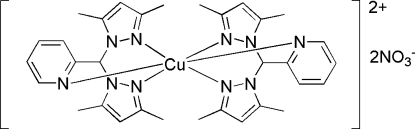


## Experimental

### 

#### Crystal data

[Cu(C_16_H_19_N_5_)_2_](NO_3_)_2_
*M* *_r_* = 750.28
Monoclinic, 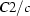

*a* = 24.819 (6) Å
*b* = 10.918 (3) Å
*c* = 17.592 (4) Å
β = 132.348 (2)°
*V* = 3523.0 (14) Å^3^
*Z* = 4
Mo *K*α radiation
μ = 0.68 mm^−1^
*T* = 296 K
0.23 × 0.22 × 0.16 mm

#### Data collection

Bruker APEXII CCD diffractometer
Absorption correction: multi-scan (*SADABS*; Bruker, 2008 ▶) *T*_min_ = 0.859, *T*_max_ = 0.899
12411 measured reflections
3266 independent reflections
2601 reflections with *I* > 2σ(*I*)
*R*_int_ = 0.022

#### Refinement

*R*[*F* ^2^ > 2σ(*F* ^2^)] = 0.036
*wR*(*F* ^2^) = 0.104
*S* = 1.07
3266 reflections
235 parameters
H-atom parameters constrained
Δρ_max_ = 0.37 e Å^−3^
Δρ_min_ = −0.32 e Å^−3^

### 

Data collection: *APEX2* (Bruker, 2008 ▶); cell refinement: *SAINT* (Bruker, 2008 ▶); data reduction: *SAINT*; program(s) used to solve structure: *SHELXS97* (Sheldrick, 2008 ▶); program(s) used to refine structure: *SHELXL97* (Sheldrick, 2008 ▶); molecular graphics: *SHELXTL* (Sheldrick, 2008 ▶); software used to prepare material for publication: *SHELXTL*.

## Supplementary Material

Crystal structure: contains datablock(s) I, global. DOI: 10.1107/S1600536811045636/ff2036sup1.cif

Structure factors: contains datablock(s) I. DOI: 10.1107/S1600536811045636/ff2036Isup2.hkl

Additional supplementary materials:  crystallographic information; 3D view; checkCIF report

## Figures and Tables

**Table 1:** Hydrogen-bond geometry (Å, °)

*D*—H⋯*A*	*D*—H	H⋯*A*	*D*⋯*A*	*D*—H⋯*A*
C2—H2⋯O3^i^	0.93	2.39	3.245 (4)	153
C8—H8⋯O2^ii^	0.93	2.46	3.338 (4)	158
C15—H15c⋯O2^iii^	0.96	2.29	3.202 (6)	159

Symmetry codes: (i) 
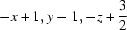
; (ii) 
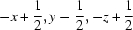
; (iii) 
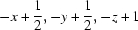
.

**Fig. 1.**
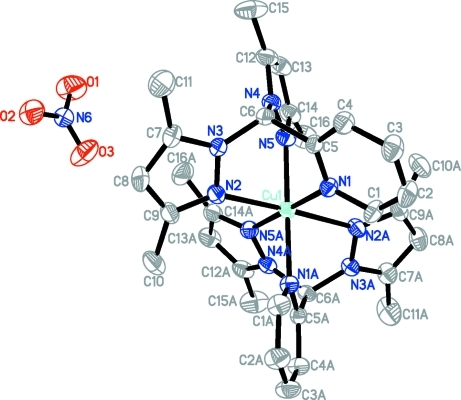
The structure of the title compound. Displacement ellipsoids are drawn at the 30% probability level. H atoms have been omitted for clarity. Symmetry code: (A)= 1 - x, y, 3/2 - z.

**Fig. 2.**
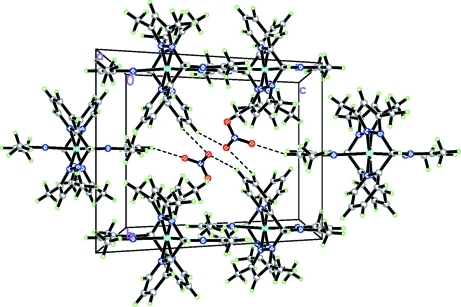
The crystal packing of the title compound, viewed along the a axis. Weak interaction among the molecules are shown as dashed lines.

## supplementary crystallographic information

### Comment

The rigid ligand with pyrazole is one of the most desirable ligand to biologists and bioinorganic chemists for specific performance, such as catalysis and fluxional behaviour (Zhang *et al.*, 2009; Otten *et al.*, 2009; Arroyo *et al.*, 2000), and also in electrochemistry (Morin *et al.* (2011)). Especially, the research field dealing with copper complexes embrace wide range of topics, such as metastasis development (Turski *et al.*, 2009; Finney *et al.*, 2009), anticancer activity (Tardito *et al.*, 2009), and other aspects of bioinorganic chemistry. In the present work, we report the synthesis and the structure of the title complex [Cu(bpz*mpy)_2_](NO_3_)_2._

An X-ray diffraction study performed on title complex [Cu(bpz*mpy)_2_](NO_3_)_2_ (Fig. 1) reveals that it crystallizes in the monoclinic system with space group *C*2/*c*. The central copper ion is six-coordinated by six nitrogen atoms from two ligands. N(1), N(1A), N(5) and N(5A) atoms form the equatorial plane with distance of Cu—N being in the range of 2.030 (2)–2.045 (2) Å, N(2) and N(2 A) atoms are in apical position with distance of Cu—N being 2.339 (2) Å [symmetry codes: (A) = 1 - *x*, *y*, 3/2 - *z*]. Consequently, the central copper ion coordination geometry can be described as a distorted octahedral coordination environment.

In the crystal, molecules are linked by weak intermolecular C—H···O interactions (Fig. 2).

### Experimental

Cu(NO_3_)_2_.3H_2_O (0.1 mmol, 24.2 mg), bpz*mpy (0.2 mmol, 56.3 mg) were dissolved in MeOH. The resulting green solution was stirred for 1 h at the ambient temperature. Blue and block crystal was obtained by evaporation after one week, and washed with methol. Yield: 47 wt%.

### Refinement

The H atoms were included in calculated positions and treated as riding atoms: C–H = 0.93- 0.98 Å, with *U*_iso_(H) = 1.2Ueq(C), *U*_iso_(H) = 1.5Ueq(Cmethyl)

### Crystal data

**Table:**

[Cu(C_16_H_19_N_5_)_2_](NO_3_)_2_	*F*(000) = 1564
*M**_r_* = 750.28	*D*_x_ = 1.415 Mg m^−^^3^
Monoclinic, *C*2/*c*	Mo *K*α radiation, λ = 0.71073 Å
Hall symbol: -C 2yc	Cell parameters from 4634 reflections
*a* = 24.819 (6) Å	θ = 2.2–25.8°
*b* = 10.918 (3) Å	µ = 0.68 mm^−^^1^
*c* = 17.592 (4) Å	*T* = 296 K
β = 132.348 (2)°	Block, blue
*V* = 3523.0 (14) Å^3^	0.23 × 0.22 × 0.16 mm
*Z* = 4	

### Data collection

**Table:**

Bruker APEXII CCD diffractometer	3266 independent reflections
Radiation source: fine-focus sealed tube	2601 reflections with *I* > 2σ(*I*)
graphite	*R*_int_ = 0.022
φ and ω scans	θ_max_ = 25.5°, θ_min_ = 2.2°
Absorption correction: multi-scan (*SADABS*; Bruker, 2008)	*h* = −30→29
*T*_min_ = 0.859, *T*_max_ = 0.899	*k* = −13→13
12411 measured reflections	*l* = −20→21

### Refinement

**Table:**

Refinement on *F*^2^	Primary atom site location: structure-invariant direct methods
Least-squares matrix: full	Secondary atom site location: difference Fourier map
*R*[*F*^2^ > 2σ(*F*^2^)] = 0.036	Hydrogen site location: inferred from neighbouring sites
*wR*(*F*^2^) = 0.104	H-atom parameters constrained
*S* = 1.07	*w* = 1/[σ^2^(*F*_o_^2^) + (0.0412*P*)^2^ + 5.0393*P*] where *P* = (*F*_o_^2^ + 2*F*_c_^2^)/3
3266 reflections	(Δ/σ)_max_ < 0.001
235 parameters	Δρ_max_ = 0.37 e Å^−^^3^
0 restraints	Δρ_min_ = −0.32 e Å^−^^3^

### Special details

**Table:**

Geometry. All e.s.d.'s (except the e.s.d. in the dihedral angle between two l.s. planes) are estimated using the full covariance matrix. The cell e.s.d.'s are taken into account individually in the estimation of e.s.d.'s in distances, angles and torsion angles; correlations between e.s.d.'s in cell parameters are only used when they are defined by crystal symmetry. An approximate (isotropic) treatment of cell e.s.d.'s is used for estimating e.s.d.'s involving l.s. planes.
Refinement. Refinement of *F*^2^ against ALL reflections. The weighted *R*-factor *wR* and goodness of fit *S* are based on *F*^2^, conventional *R*-factors *R* are based on *F*, with *F* set to zero for negative *F*^2^. The threshold expression of *F*^2^ > σ(*F*^2^) is used only for calculating *R*-factors(gt) *etc*. and is not relevant to the choice of reflections for refinement. *R*-factors based on *F*^2^ are statistically about twice as large as those based on *F*, and *R*- factors based on ALL data will be even larger.

### Fractional atomic coordinates and isotropic or equivalent isotropic displacement parameters (Å^2^)

**Table:**

	*x*	*y*	*z*	*U*_iso_*/*U*_eq_	
Cu1	0.5000	−0.01320 (4)	0.7500	0.03860 (15)	
C1	0.48707 (15)	−0.2377 (2)	0.8338 (2)	0.0508 (7)	
H1	0.5351	−0.2502	0.8646	0.061*	
C2	0.45590 (17)	−0.3194 (3)	0.8536 (2)	0.0595 (8)	
H2	0.4828	−0.3849	0.8980	0.071*	
C3	0.38457 (16)	−0.3035 (3)	0.8069 (2)	0.0598 (8)	
H3	0.3621	−0.3586	0.8183	0.072*	
C4	0.34694 (15)	−0.2046 (2)	0.7430 (2)	0.0492 (6)	
H4	0.2985	−0.1920	0.7102	0.059*	
C5	0.38190 (13)	−0.1246 (2)	0.72802 (18)	0.0368 (5)	
C6	0.34211 (13)	−0.0120 (2)	0.66153 (19)	0.0389 (5)	
H6	0.2941	−0.0109	0.6403	0.047*	
C7	0.26736 (15)	−0.0142 (3)	0.4692 (2)	0.0554 (7)	
C8	0.28709 (17)	−0.0194 (3)	0.4136 (2)	0.0611 (8)	
H8	0.2559	−0.0196	0.3423	0.073*	
C9	0.36269 (16)	−0.0244 (2)	0.4835 (2)	0.0498 (7)	
C10	0.4118 (2)	−0.0301 (4)	0.4627 (3)	0.0755 (10)	
H10A	0.4604	−0.0078	0.5237	0.113*	
H10B	0.3947	0.0257	0.4082	0.113*	
H10C	0.4119	−0.1119	0.4427	0.113*	
C11	0.19391 (19)	−0.0068 (5)	0.4360 (3)	0.1009 (15)	
H11A	0.1859	−0.0785	0.4588	0.151*	
H11B	0.1570	−0.0018	0.3623	0.151*	
H11C	0.1916	0.0647	0.4655	0.151*	
C12	0.35339 (18)	0.1970 (3)	0.7337 (2)	0.0553 (7)	
C13	0.4112 (2)	0.2737 (3)	0.7987 (3)	0.0666 (9)	
H13	0.4104	0.3487	0.8230	0.080*	
C14	0.47163 (17)	0.2200 (2)	0.8223 (2)	0.0540 (7)	
C15	0.2759 (2)	0.2089 (3)	0.6840 (4)	0.0882 (12)	
H15A	0.2455	0.2171	0.6110	0.132*	
H15B	0.2705	0.2800	0.7105	0.132*	
H15C	0.2617	0.1373	0.6985	0.132*	
C16	0.54737 (19)	0.2688 (3)	0.8906 (3)	0.0773 (10)	
H16A	0.5811	0.2021	0.9172	0.116*	
H16B	0.5580	0.3130	0.9465	0.116*	
H16C	0.5518	0.3228	0.8520	0.116*	
N1	0.45132 (11)	−0.14066 (18)	0.77218 (15)	0.0392 (5)	
N2	0.38955 (12)	−0.0231 (2)	0.57884 (16)	0.0463 (5)	
N3	0.33024 (11)	−0.01645 (19)	0.56902 (16)	0.0416 (5)	
N4	0.37949 (12)	0.09956 (18)	0.71912 (16)	0.0420 (5)	
N5	0.45224 (12)	0.11305 (19)	0.77316 (17)	0.0448 (5)	
N6	0.35109 (14)	0.5779 (2)	0.41294 (19)	0.0512 (6)	
O1	0.35080 (16)	0.6715 (3)	0.4505 (2)	0.1132 (11)	
O2	0.29475 (13)	0.5446 (3)	0.3289 (2)	0.0896 (8)	
O3	0.40686 (15)	0.5216 (2)	0.4542 (2)	0.1047 (10)	

### Atomic displacement parameters (Å^2^)

**Table:**

	*U*^11^	*U*^22^	*U*^33^	*U*^12^	*U*^13^	*U*^23^
Cu1	0.0389 (3)	0.0372 (2)	0.0438 (3)	0.000	0.0295 (2)	0.000
C1	0.0402 (15)	0.0482 (15)	0.0480 (16)	0.0088 (12)	0.0232 (14)	0.0131 (12)
C2	0.0570 (19)	0.0490 (16)	0.0591 (19)	0.0058 (13)	0.0337 (16)	0.0185 (14)
C3	0.0576 (19)	0.0546 (17)	0.0637 (19)	−0.0056 (14)	0.0394 (17)	0.0134 (14)
C4	0.0440 (15)	0.0497 (15)	0.0526 (16)	−0.0009 (12)	0.0320 (14)	0.0070 (13)
C5	0.0368 (13)	0.0370 (12)	0.0341 (13)	0.0017 (10)	0.0229 (12)	0.0016 (10)
C6	0.0371 (13)	0.0431 (13)	0.0409 (13)	0.0054 (11)	0.0280 (12)	0.0068 (11)
C7	0.0431 (16)	0.0669 (18)	0.0400 (15)	0.0112 (14)	0.0215 (14)	0.0093 (13)
C8	0.0593 (19)	0.075 (2)	0.0334 (14)	0.0083 (16)	0.0248 (15)	0.0067 (14)
C9	0.0621 (18)	0.0511 (15)	0.0426 (15)	0.0026 (13)	0.0378 (15)	0.0022 (12)
C10	0.085 (2)	0.103 (3)	0.061 (2)	0.004 (2)	0.059 (2)	0.0016 (19)
C11	0.0434 (19)	0.176 (5)	0.057 (2)	0.017 (2)	0.0226 (18)	0.004 (2)
C12	0.078 (2)	0.0428 (15)	0.078 (2)	0.0118 (14)	0.0658 (19)	0.0078 (14)
C13	0.108 (3)	0.0402 (15)	0.096 (3)	−0.0011 (17)	0.086 (2)	−0.0072 (16)
C14	0.076 (2)	0.0427 (15)	0.0636 (19)	−0.0079 (14)	0.0553 (18)	−0.0085 (13)
C15	0.093 (3)	0.062 (2)	0.147 (4)	0.0158 (19)	0.096 (3)	0.001 (2)
C16	0.088 (3)	0.062 (2)	0.090 (3)	−0.0267 (18)	0.063 (2)	−0.0329 (18)
N1	0.0345 (11)	0.0405 (11)	0.0383 (12)	0.0020 (9)	0.0227 (10)	0.0039 (9)
N2	0.0425 (12)	0.0615 (14)	0.0384 (12)	0.0045 (10)	0.0286 (11)	0.0039 (10)
N3	0.0368 (11)	0.0503 (12)	0.0366 (11)	0.0080 (9)	0.0243 (10)	0.0077 (9)
N4	0.0501 (13)	0.0391 (11)	0.0494 (13)	0.0049 (9)	0.0386 (12)	0.0045 (9)
N5	0.0505 (13)	0.0415 (12)	0.0508 (13)	−0.0030 (10)	0.0374 (12)	−0.0043 (10)
N6	0.0515 (15)	0.0467 (13)	0.0554 (15)	−0.0091 (11)	0.0361 (14)	−0.0065 (11)
O1	0.103 (2)	0.098 (2)	0.116 (2)	−0.0088 (17)	0.065 (2)	−0.0504 (18)
O2	0.0583 (15)	0.0995 (19)	0.0702 (16)	−0.0095 (14)	0.0268 (14)	−0.0291 (14)
O3	0.0630 (16)	0.0806 (18)	0.098 (2)	0.0121 (14)	0.0247 (16)	−0.0141 (15)

### Geometric parameters (Å, °)

**Table:**

Cu1—N5^i^	2.030 (2)	C9—C10	1.491 (4)
Cu1—N5	2.030 (2)	C10—H10A	0.9600
Cu1—N1	2.045 (2)	C10—H10B	0.9600
Cu1—N1^i^	2.045 (2)	C10—H10C	0.9600
Cu1—N2^i^	2.339 (2)	C11—H11A	0.9600
Cu1—N2	2.339 (2)	C11—H11B	0.9600
C1—N1	1.337 (3)	C11—H11C	0.9600
C1—C2	1.370 (4)	C12—N4	1.358 (3)
C1—H1	0.9300	C12—C13	1.363 (4)
C2—C3	1.371 (4)	C12—C15	1.488 (4)
C2—H2	0.9300	C13—C14	1.388 (4)
C3—C4	1.374 (4)	C13—H13	0.9300
C3—H3	0.9300	C14—N5	1.334 (3)
C4—C5	1.376 (3)	C14—C16	1.490 (4)
C4—H4	0.9300	C15—H15A	0.9600
C5—N1	1.341 (3)	C15—H15B	0.9600
C5—C6	1.516 (3)	C15—H15C	0.9600
C6—N3	1.446 (3)	C16—H16A	0.9600
C6—N4	1.451 (3)	C16—H16B	0.9600
C6—H6	0.9800	C16—H16C	0.9600
C7—N3	1.352 (3)	N2—N3	1.363 (3)
C7—C8	1.359 (4)	N4—N5	1.369 (3)
C7—C11	1.495 (5)	N6—O3	1.211 (3)
C8—C9	1.388 (4)	N6—O2	1.219 (3)
C8—H8	0.9300	N6—O1	1.220 (3)
C9—N2	1.324 (3)		
			
N5^i^—Cu1—N5	94.48 (12)	C9—C10—H10C	109.5
N5^i^—Cu1—N1	179.57 (9)	H10A—C10—H10C	109.5
N5—Cu1—N1	85.63 (8)	H10B—C10—H10C	109.5
N5^i^—Cu1—N1^i^	85.63 (8)	C7—C11—H11A	109.5
N5—Cu1—N1^i^	179.57 (9)	C7—C11—H11B	109.5
N1—Cu1—N1^i^	94.26 (12)	H11A—C11—H11B	109.5
N5^i^—Cu1—N2^i^	87.32 (8)	C7—C11—H11C	109.5
N5—Cu1—N2^i^	96.28 (8)	H11A—C11—H11C	109.5
N1—Cu1—N2^i^	93.08 (8)	H11B—C11—H11C	109.5
N1^i^—Cu1—N2^i^	83.31 (8)	N4—C12—C13	105.8 (3)
N5^i^—Cu1—N2	96.28 (8)	N4—C12—C15	123.3 (3)
N5—Cu1—N2	87.32 (8)	C13—C12—C15	130.8 (3)
N1—Cu1—N2	83.31 (8)	C12—C13—C14	107.9 (3)
N1^i^—Cu1—N2	93.08 (8)	C12—C13—H13	126.1
N2^i^—Cu1—N2	174.72 (11)	C14—C13—H13	126.1
N1—C1—C2	122.8 (3)	N5—C14—C13	109.3 (3)
N1—C1—H1	118.6	N5—C14—C16	123.0 (3)
C2—C1—H1	118.6	C13—C14—C16	127.7 (3)
C1—C2—C3	119.2 (3)	C12—C15—H15A	109.5
C1—C2—H2	120.4	C12—C15—H15B	109.5
C3—C2—H2	120.4	H15A—C15—H15B	109.5
C2—C3—C4	118.8 (3)	C12—C15—H15C	109.5
C2—C3—H3	120.6	H15A—C15—H15C	109.5
C4—C3—H3	120.6	H15B—C15—H15C	109.5
C3—C4—C5	119.2 (3)	C14—C16—H16A	109.5
C3—C4—H4	120.4	C14—C16—H16B	109.5
C5—C4—H4	120.4	H16A—C16—H16B	109.5
N1—C5—C4	122.4 (2)	C14—C16—H16C	109.5
N1—C5—C6	117.9 (2)	H16A—C16—H16C	109.5
C4—C5—C6	119.7 (2)	H16B—C16—H16C	109.5
N3—C6—N4	111.33 (19)	C1—N1—C5	117.7 (2)
N3—C6—C5	111.97 (19)	C1—N1—Cu1	122.61 (17)
N4—C6—C5	111.4 (2)	C5—N1—Cu1	119.60 (15)
N3—C6—H6	107.3	C9—N2—N3	105.1 (2)
N4—C6—H6	107.3	C9—N2—Cu1	141.70 (19)
C5—C6—H6	107.3	N3—N2—Cu1	112.94 (15)
N3—C7—C8	105.9 (3)	C7—N3—N2	111.6 (2)
N3—C7—C11	123.1 (3)	C7—N3—C6	130.0 (2)
C8—C7—C11	131.1 (3)	N2—N3—C6	118.4 (2)
C7—C8—C9	107.0 (3)	C12—N4—N5	111.0 (2)
C7—C8—H8	126.5	C12—N4—C6	128.9 (2)
C9—C8—H8	126.5	N5—N4—C6	119.95 (19)
N2—C9—C8	110.4 (2)	C14—N5—N4	106.0 (2)
N2—C9—C10	121.0 (3)	C14—N5—Cu1	136.0 (2)
C8—C9—C10	128.7 (3)	N4—N5—Cu1	117.58 (15)
C9—C10—H10A	109.5	O3—N6—O2	119.3 (3)
C9—C10—H10B	109.5	O3—N6—O1	121.5 (3)
H10A—C10—H10B	109.5	O2—N6—O1	119.1 (3)
			
N1—C1—C2—C3	1.4 (5)	N5—Cu1—N2—N3	−38.62 (17)
C1—C2—C3—C4	−1.0 (5)	N1—Cu1—N2—N3	47.29 (16)
C2—C3—C4—C5	−0.2 (4)	N1^i^—Cu1—N2—N3	141.22 (17)
C3—C4—C5—N1	1.3 (4)	C8—C7—N3—N2	0.0 (3)
C3—C4—C5—C6	−177.2 (3)	C11—C7—N3—N2	−179.8 (3)
N1—C5—C6—N3	67.1 (3)	C8—C7—N3—C6	179.4 (2)
C4—C5—C6—N3	−114.3 (3)	C11—C7—N3—C6	−0.4 (5)
N1—C5—C6—N4	−58.3 (3)	C9—N2—N3—C7	0.2 (3)
C4—C5—C6—N4	120.3 (2)	Cu1—N2—N3—C7	175.91 (18)
N3—C7—C8—C9	−0.2 (3)	C9—N2—N3—C6	−179.3 (2)
C11—C7—C8—C9	179.6 (4)	Cu1—N2—N3—C6	−3.6 (3)
C7—C8—C9—N2	0.3 (3)	N4—C6—N3—C7	−114.3 (3)
C7—C8—C9—C10	−179.5 (3)	C5—C6—N3—C7	120.3 (3)
N4—C12—C13—C14	−0.1 (3)	N4—C6—N3—N2	65.1 (3)
C15—C12—C13—C14	−179.6 (3)	C5—C6—N3—N2	−60.3 (3)
C12—C13—C14—N5	0.3 (3)	C13—C12—N4—N5	−0.1 (3)
C12—C13—C14—C16	−179.8 (3)	C15—C12—N4—N5	179.4 (3)
C2—C1—N1—C5	−0.4 (4)	C13—C12—N4—C6	176.1 (2)
C2—C1—N1—Cu1	176.1 (2)	C15—C12—N4—C6	−4.4 (4)
C4—C5—N1—C1	−1.0 (4)	N3—C6—N4—C12	110.1 (3)
C6—C5—N1—C1	177.6 (2)	C5—C6—N4—C12	−124.1 (3)
C4—C5—N1—Cu1	−177.61 (19)	N3—C6—N4—N5	−74.0 (3)
C6—C5—N1—Cu1	1.0 (3)	C5—C6—N4—N5	51.8 (3)
N5—Cu1—N1—C1	−133.1 (2)	C13—C14—N5—N4	−0.4 (3)
N1^i^—Cu1—N1—C1	46.47 (18)	C16—C14—N5—N4	179.8 (3)
N2^i^—Cu1—N1—C1	−37.0 (2)	C13—C14—N5—Cu1	171.5 (2)
N2—Cu1—N1—C1	139.1 (2)	C16—C14—N5—Cu1	−8.3 (5)
N5—Cu1—N1—C5	43.32 (18)	C12—N4—N5—C14	0.3 (3)
N1^i^—Cu1—N1—C5	−137.1 (2)	C6—N4—N5—C14	−176.3 (2)
N2^i^—Cu1—N1—C5	139.39 (18)	C12—N4—N5—Cu1	−173.37 (17)
N2—Cu1—N1—C5	−44.48 (18)	C6—N4—N5—Cu1	10.0 (3)
C8—C9—N2—N3	−0.4 (3)	N5^i^—Cu1—N5—C14	−40.8 (2)
C10—C9—N2—N3	179.5 (3)	N1—Cu1—N5—C14	139.7 (3)
C8—C9—N2—Cu1	−173.9 (2)	N2^i^—Cu1—N5—C14	47.0 (3)
C10—C9—N2—Cu1	5.9 (5)	N2—Cu1—N5—C14	−136.9 (3)
N5^i^—Cu1—N2—C9	40.4 (3)	N5^i^—Cu1—N5—N4	130.4 (2)
N5—Cu1—N2—C9	134.6 (3)	N1—Cu1—N5—N4	−49.17 (17)
N1—Cu1—N2—C9	−139.5 (3)	N2^i^—Cu1—N5—N4	−141.80 (17)
N1^i^—Cu1—N2—C9	−45.6 (3)	N2—Cu1—N5—N4	34.32 (17)
N5^i^—Cu1—N2—N3	−132.84 (17)		

Symmetry codes: (i) −*x*+1, *y*, −*z*+3/2.

### Hydrogen-bond geometry (Å, °)

**Table:**

*D*—H···*A*	*D*—H	H···*A*	*D*···*A*	*D*—H···*A*
C2—H2···O3^ii^	0.93	2.39	3.245 (4)	153
C8—H8···O2^iii^	0.93	2.46	3.338 (4)	158
C15—H15c···O2^iv^	0.96	2.29	3.202 (6)	159

Symmetry codes: (ii) −*x*+1, *y*−1, −*z*+3/2; (iii) −*x*+1/2, *y*−1/2, −*z*+1/2; (iv) −*x*+1/2, −*y*+1/2, −*z*+1.
